# Supplementary material for: Submicronic Filtering Media Based on Electrospun Recycled PET Nanofibers: Development, Characterization, and Method to Manufacture Surgical Masks
Source: Nanomaterials (Basel). 2022 Mar 11;12(6):925. doi: 10.3390/nano12060925 (PMC8952864; doi:10.3390/nano12060925)
Supplement: Supplementary file 1 [file nanomaterials-12-00925-s001.zip › nanomaterials-1624423-supplementary.pdf]

Submicronic Filtering Media Based on Electrospun Recycled PET Nanofibers: Development, Characterization and Method to Manufacture a Surgical Mask

Marta Baselga-Lahoz[1], Cristina Yus[1,2], Manuel Arruebo[1,2,3,4], Víctor Sebastián[1,2,3,4], Silvia Irusta[1,2,3,4], Santiago Jiménez[5]

[1]Institute for Health Research Aragon (IIS Aragón), 50009 Zaragoza, Spain

[2]Instituto de Nanociencia y Materiales de Aragon (INMA), CSIC – University of Zaragoza, 50009 Zaragoza, Spain

[3]Department of Chemical Engineering, University of Zaragoza, Campus Río Ebro – I+D Building, 50018 Zaragoza, Spain

[4]Networking Research Center on Bioengineering, Biomaterials and Nanomedicine, CIBER-BBN, 28029 Madrid, Spain

[5]Laboratorio de Fluidodinámica y Tecnologías de la Combustión (Liftec), CSIC – University of Zaragoza, 50018 Zaragoza, Spain

Supplementary Material Section

Retention efficiency and pressure drop determination

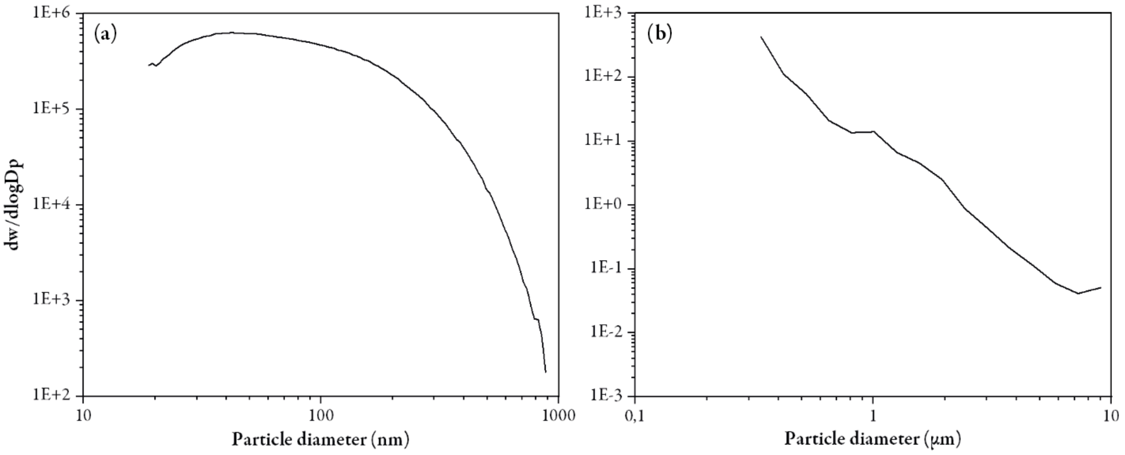

Figure S1. Particle concentration distribution depending on their diameter used for the efficiency determination measurements in the range (a) 0.01 - 0.5 µm (SMPS) and (b) > 0.5 µm (OPS).

Homogeneity across the fabric

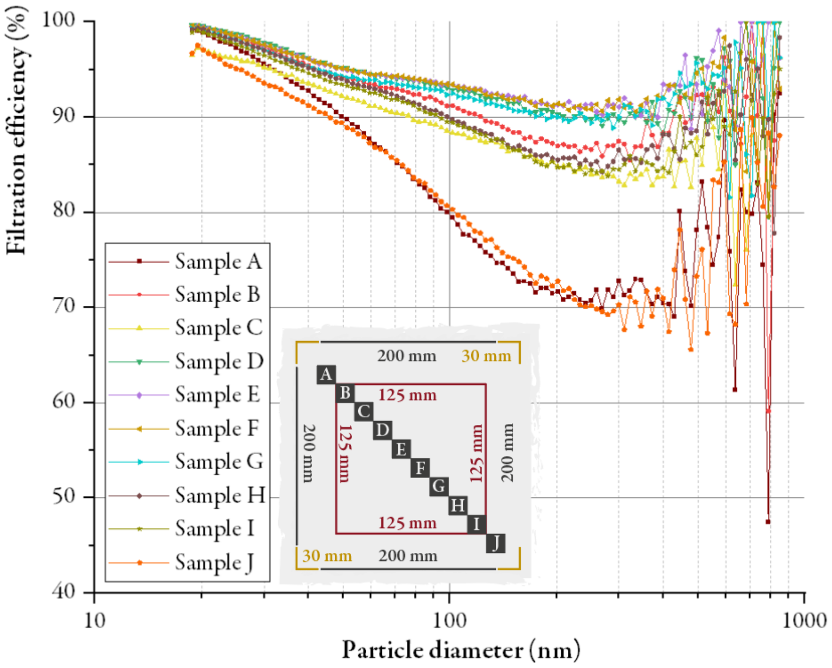

Figure S2. Evaluation of the homogeneity of a sample of filter fabric.

Drying of fabric samples

Figure S2 shows the morphological deformation of the fibers after being exposed to a temperature of 80ºC. The deterioration of the fibers is evident, finding notches on the surface of the fibers and a morphological change that translates into a greater porosity of the material.

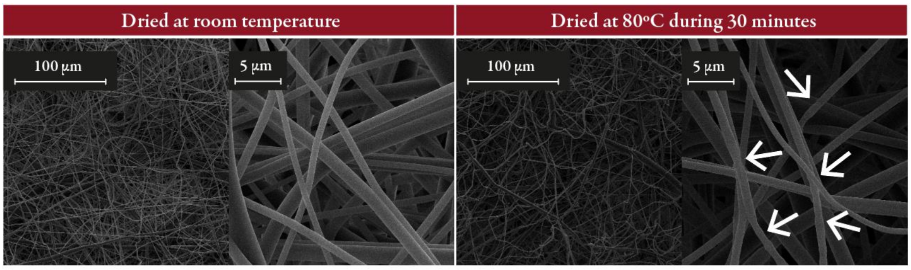

Figure S3. SEM images of fibers of the morphological deformation of PET fibers after being dried at 80°C for 30 minutes.
